# Addressing the Protease Bias in Quantitative Proteomics

**DOI:** 10.1021/acs.jproteome.2c00491

**Published:** 2022-08-31

**Authors:** Jakob Woessmann, David Kotol, Andreas Hober, Mathias Uhlén, Fredrik Edfors

**Affiliations:** † Science for Life Laboratory, KTH—Royal Institute of Technology, SE-171 65 Solna, Sweden; ‡ Department of Protein Science, KTH—Royal Institute of Technology, SE-106 91 Stockholm, Sweden

**Keywords:** multiple proteases, SRM, targeted proteomics, absolute quantification, plasma proteomics

## Abstract

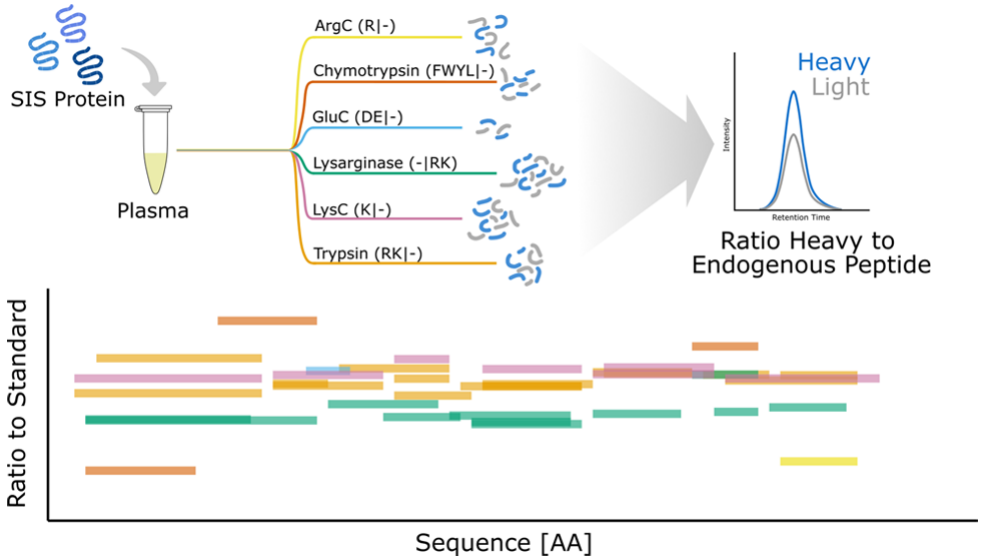

Protein quantification strategies using multiple proteases have been shown to deliver poor interprotease accuracy in label-free mass spectrometry experiments. By utilizing six different proteases with different cleavage sites, this study explores the protease bias and its effect on accuracy and precision by using recombinant protein standards. We established 557 SRM assays, using a recombinant protein standard resource, toward 10 proteins in human plasma and determined their concentration with multiple proteases. The quantified peptides of these plasma proteins spanned 3 orders of magnitude (0.02–70 μM). In total, 60 peptides were used for absolute quantification and the majority of the peptides showed high robustness. The retained reproducibility was achieved by quantifying plasma proteins using spiked stable isotope standard recombinant proteins in a targeted proteomics workflow.

## Introduction

Trypsin has for decades been the predominant protease for protein digestion in the field of bottom-up proteomics.^1−3^ The advantages of trypsin over other enzymes are well known, and it has become the protease of choice due to its ability to enhance peptide detection by generating C-terminal charges and a peptide repertoire, which consists of relatively short peptides that are suitable for analysis using liquid chromatography (LC) tandem mass spectrometry (MS/MS).^4^ However, other proteases have recently been suggested and introduced to the bottom-up proteomics workflow to compensate for the downsides of tryptic digests, such as the overall shorter tryptic peptide repertoire.^5,6^ Therefore, valuable work has been made to utilize alternative proteases to improve the overall sequence coverage of proteomes and to increase the peptide repertoire of specific proteins with poor trypsin compatibility.^5,6^ These approaches have mainly focused on expanding the proteome coverage in data-dependent acquisition and data-independent acquisition experiments^7,8^ or on the detection of posttranslational modifications.^9^ In targeted proteomics assays, it was shown that the combination of trypsin, LysN, and chymotrypsin could be used to extend protein coverage.^10^ However, Peng et al. and Giansanti et al. have shown a poor correlation in label-free absolute quantification between different proteases.^9,11^ The results from these two studies showed that the label-free intensity or spectral counts of the same sample digested by different proteases delivered poor correlation between samples when digested by different proteases. Interestingly, these studies focused solely on label-free quantification and did not address the use of stable isotope-labeled standards, which has the potential to overcome the quantitative bias, in regard to alternative proteases, and would allow for comparison across proteases on an absolute scale.

Targeted proteomics provides high sensitivity and specificity and has become very popular thanks to its capability to deliver both precise and robust protein quantification.^12^ Targeted proteomics workflows are often based on triple quadrupole instrumentation, which is associated with a front-heavy approach that includes time-consuming steps such as assay generation and validation of each peptide. This makes protein quantification very labor-intensive, and the cost of developing assays is further increased by the need for synthetic and stable isotope-labeled peptides. This approach makes it hard to alternate between quantitative peptides, and the additional cost of replacing trypsin with other proteases more suitable for specific proteins may seem overwhelming. The change would not only increase the cost but the protease specificity would also deem most of the peptides in a trypsin-based synthetic peptide library obsolete. The latter can be overcome by using recombinant full-length proteins or protein fragments where the peptide repertoire is generated with high flexibility by simply alternating the protease. Recombinant protein standards often provide a limited amount of tryptic peptides that can be used for absolute quantification. Therefore, it can be challenging to confirm the quantification results solely relying on tryptic peptides. If it would be possible to quantify peptides of alternative proteases without a bias, the tryptic peptide quantification could be validated by the extended peptide repertoire. Furthermore, digestion with alternative proteases would control for digestion-dependent biases in quantitative experiments. Therefore, multiple proteases could provide a simple and accessible strategy for the targeted proteomics community to verify the accuracy of peptide quantifications while extending the peptide quantitative repertoire beyond trypsin.

Here, we introduce a comprehensive multiple protease approach to the quantitative analysis of plasma proteins based on recombinant protein standards quantified by LC selective reaction monitoring (SRM) mass spectrometry (MS).^13^ We demonstrate that the combined peptide repertoire from the six proteases ArgC, GluC, chymotrypsin, lysarginase, LysC, and trypsin leads to nearly complete coverage of the used recombinant protein standards and provides a wide peptide variety, which can be monitored through a standardized and easy-to-follow assay development workflow. Further, we demonstrate consistent quantification results in up to six different proteases for multiple plasma proteins. Moreover, miscleaved peptides and fully digested peptides show no significant differences when quantified using recombinant protein standards.

## Methods

### Sample Preparation and SRM Analysis

Recombinant protein standards in the form of protein epitope signature tags (PrESTs, up to 149 amino acids (AAs) long) and the stable isotope standard (SIS) PrESTs (^13^C and ^15^N labeled) were developed within the Human Protein Atlas, as previously described.^1^ To establish SRM assays, PrESTs were digested individually by six different proteases. PrESTs were reduced by dithiothreitol (DTT, 10 mM, 30 min, 56 °C) followed by alkylation with 2-chloroacetamide (CAA, 50 mM, 30 min, room temperature (RT)) in the dark. For the digestion with lysarginase (Sigma-Aldrich), endoproteinase ArgC (Roche), and chymotrypsin (Thermo Scientific), CaCl_2_ was added to a final concentration of 10 mM. The volume of the trypsin (Thermo Scientific), GluC (Thermo Scientific), and LysC (Wako) digestion was adjusted by 1× PBS. Digestion was performed in a 1:20 enzyme to protein ratio (E/P) overnight and was quenched by the addition of trifluoroacetic acid (TFA) to a final concentration of 0.5%.

Plasma samples from three healthy males and two females were pooled. For the absolute quantification, the plasma was spiked with a pool of SIS-PrESTs close to endogenous protein levels (Table 1). The spike-in levels were determined previously in an iterative spike-in adjustment process.^1^ The mixture was diluted 30 times with 1% sodium deoxycholate (SDC) and 1 M urea. Samples were reduced and alkylated as described above. SDC was diluted to 0.1%, and the amount corresponding to 1 μL of plasma was digested with each protease. For the digestion with lysarginase, ArgC, and chymotrypsin, CaCl_2_ was added to a final concentration of 10 mM. Digestion with ArgC (1:50 E/P), chymotrypsin (1:50 E/P), GluC (1:50 E/P), LysC (1:50 E/P), lysarginase (1:20 E/P), and trypsin (1:50 E/P) was each performed in triplicate overnight. The digestion was quenched by the addition of TFA to a final concentration of 0.5%. Each digest was desalted by means of a 6-layer C18 StageTips prepared in-house, as described by Kotol et al.^14^ The eluate was vacuum-dried at 45 °C and stored at −20 °C until MS analysis.

**Table 1:** SIS-PrESTs Included in the SRM Assay Development and Amount Spiked For Plasma Protein Quantification

gene	uniprot accession	plasma spike-in [μM]	PrEST seq [AA]
APMAP	Q9HDC9	0.014	PLSFKEPPLLLGVLHPNTKLRQAERLFENQLVGPESIAHIGDVMFTGTADGRVVKLENGEIETIAR
			FGSGPCKTRDDEPVCGRPLGIRAGPNGTLFVADAYKGLFEVNPWKREVKLLL
APOA1	P02647	33.298	SKLREQLGPVTQEFWDNLEKETEGLRQEMSKDLEEVKAKVQPYLDDFQKKWQEEMELYRQKV
			EPLRAELQEGARQKLHELQEKL
APOL1	O14791	0.347	SNFLSLAGNTYQLTRGIGKDIRALRRARANLQSVPHASASRPRVTEPISAESGEQVERVNEPSILE
			MSRGVKLTDVAPVSFFLVLDVVYLVYESKHLHEGAKSETAEELKKVAQELEEKLNILNN
CRTAC1	Q9NQ79	0.055	VVTDFDGDGMLDLILSHGESMAQPLSVFRGNQGFNNNWLRVVPRTRFGAFARGAKVVLYTKKS
			GAHLRIIDGGSGYLCEMEPVAHFGLGKDEASSVEVTWPDGKMVSRNVASGEMNSVLEILYPRDE
			DTLQDPAP
F10	P00742		EVEVVIKHNRFTKETYDFDIAVLRLKTPITFRMNVAPACLPERDWAESTLMTQKTGIVSGFGRTHE
			KGRQSTRLKMLEVPYVDRNSCKLSSSFIITQ
FGA	P02671	14.926	GHWTSESSVSGSTGQWHSESGSFRPDSPGSGNARPNNPDWGTFEEVSGNVSPGTRREYHTE
			KLVTSKGDKELRTGKEKVTSGSTTTTRRSCSKTVTKT
FGG	P02679		MIDAATLKSRKMLEEIMKYEASILTHDSSIRYLQEIYNSNNQKIVNLKEKVAQLEAQCQEPCKDTVQ
			IHDITGKDCQDIANKGAKQSGLYFIKPLKANQQFLVYCEIDGSGNGWTVFQKRLDGSVDFKKNWI
			QYKEGFGHLSPTGTTEF
GLIPR2	Q9H4G4	0.009	GKSASKQFHNEVLKAHNEYRQKHGVPPLKLCKNLNREAQQYSEALASTRILKHSPESSRGQCGE
			NLAWASYD
IGF2	P01344		TLQFVCGDRGFYFSRPASRVSRRSRGIVEECCFRSCDLALLETYCATPAKSERDVSTPPTVLPDN
			FPRYPVGKFFQYDTWKQSTQRLRRGLPALLRARRGHVLAKELEAFREAKRHRPLIALPTQD
TGFBI	Q15582	0.072	NREGVYTVFAPTNEAFRALPPRERSRLLGDAKELANILKYHIGDEILVSGGIGALVRLKSLQGDKLE
			VSLKNNVVSVNKEPVAEPDIMATNGVVHVITNVLQPPANRPQERGDELADSALEIFKQAS

### Liquid Chromatography and Mass Spectrometry Setup

Quantification and assay development were performed on an Ultimate 3000 nano-LC (Thermo Fisher Scientific) connected to an EASY-Spray ion source and a TSQ Altis (Thermo Fisher Scientific) mass spectrometer. Samples were loaded on an Acclaim PepMap 100 trap column (75 μm × 2 cm, C18, 3 μm, 100 Å, Thermo Scientific) and washed for 0.75 min at 15 μL/min with 99% solvent A (3% acetonitrile, 0.1% formic acid (FA), H_2_O). The peptides were separated using an analytical PepMap RSLC C18 column (150 μm × 15 cm, 2 μm, 100 Å, Thermo Fisher Scientific). Peptides were eluted at a linear gradient of 1–40% solvent B (95% acetonitrile, 0.1% FA) during assay development and a linear gradient of 1–30% solvent B during protein quantification. The flow rate was set to 3 μL/min over 9.25 min during assay development and over 29.25 min during protein quantification. The columns were washed three times for 30 s with 95% solvent B followed by 1% solvent B. The columns were equilibrated for 1.4 min with 1% solvent B. The total turnaround time with sample loading, analysis, and re-equilibration was 15 min for method development and 35 min for plasma quantification. The column oven temperature was maintained at 40 °C, the analytical column was maintained at 60 °C, and the autosampler temperature was maintained at 10 °C.

### SRM Assay Development

PrESTs were separately *in silico-*digested by ArgC, GluC, LysC, lysarginase, chymotrypsin, and trypsin, generating a sequence library of all possible peptides (5–25 AA) including single miscleavages in Skyline^15^ (version 20.2.1.404). Precursor charge states of +2, +3, and +4 were included. Transition lists containing mass-to-charge ratios of all theoretical peptides together with at least 3 AA long theoretical *b*- and *y*-ions were exported. The transition lists were used for the unscheduled SRM method with a dwell time of 0.5 ms. The resulting raw files were investigated in Skyline, and all identified chromatographic events were reanalyzed in a scheduled method with a 1 min retention time window and a dwell time of over 1 ms. Only chromatographic events with a minimum of three coeluting transitions were selected. The top 20 interference-free transitions of the highest precursor charge state were selected by peak area rank by Skyline, and their collision energy was optimized in steps of ±5 V. The final methods were verified on a 29.25 min gradient, and the top 10 transitions were selected by peak area rank with a preference for larger product ions. Based on these peptides, a library was curated. Peptide retention times were mapped in the plasma background by spiking digested PrESTs directly to a 10 μg plasma digest. Peptides that were not detected in plasma digest or had a library dot product (dotp) value below 0.9 were not considered for the absolute quantification. The validated peptides and transitions were quantified in blood plasma using spiked-in SIS-PrESTs on a 29.25 min linear gradient with 5 min retention time windows and a minimum dwell time of 1.8 ms in triplicate digestion and injection replicates.

### Data Processing

LC-SRM/MS raw data from the absolute quantification were manually revised in Skyline. The top 5 transitions, based on the highest peak area, were selected with Skyline refine functions with a preference for larger product ions. Peptides with a minimum of three transitions were included. The Uniprot human canonical proteome (UP000005640, 20,588 entries, retrieved Jan 14, 2022) was set as the background proteome, and uniqueness was enforced at the protein level.

All peptide data points with a dotp value below 0.9 and a dot product light to heavy (rdotp) below 0.9 were excluded. Digestion replicates of peptides with a CV of the ratio to standard between the injections of above 20% were excluded. Additionally, replicates with only one injection passing this criterion were excluded. Absolute quantification of plasma proteins was performed by multiplication of the ratio to standard with spiked-in concentrations of SIS-PrESTs. SIS-PrEST quantification was previously performed, as described by Hober et al.^16^

### Data Availability

Raw data and Skyline documents of method development and quantification are available on Panorama Public^17^ (https://panoramaweb.org/multiple_proteases.url) with the ProteomeXchange identifier PXD033574.

## Results and Discussion

**Figure 1:**
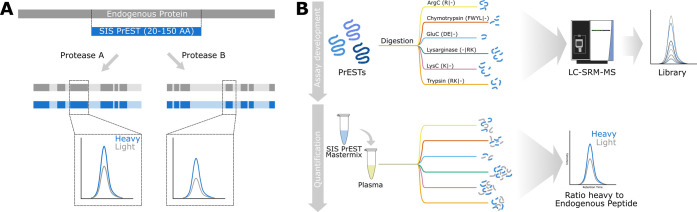
Targeted proteomics assay development using six proteases with different peptide repertoires. (A) SIS-PrESTs represent 20–150 AA sequences of endogenous proteins and can be digested by different proteases. Therefore, they can be used as heavy standards regardless of protease specificity. (B) Workflow to establish quantitative plasma proteomics SRM assays based on PrEST peptides. SRM assays for 10 PrESTs and 6 proteases were developed. Plasma proteins were quantified using spiked SIS-PrESTs and the developed assays. The plasma protein levels were quantified based on the ratio of heavy to light peptides.

**Figure 2:**
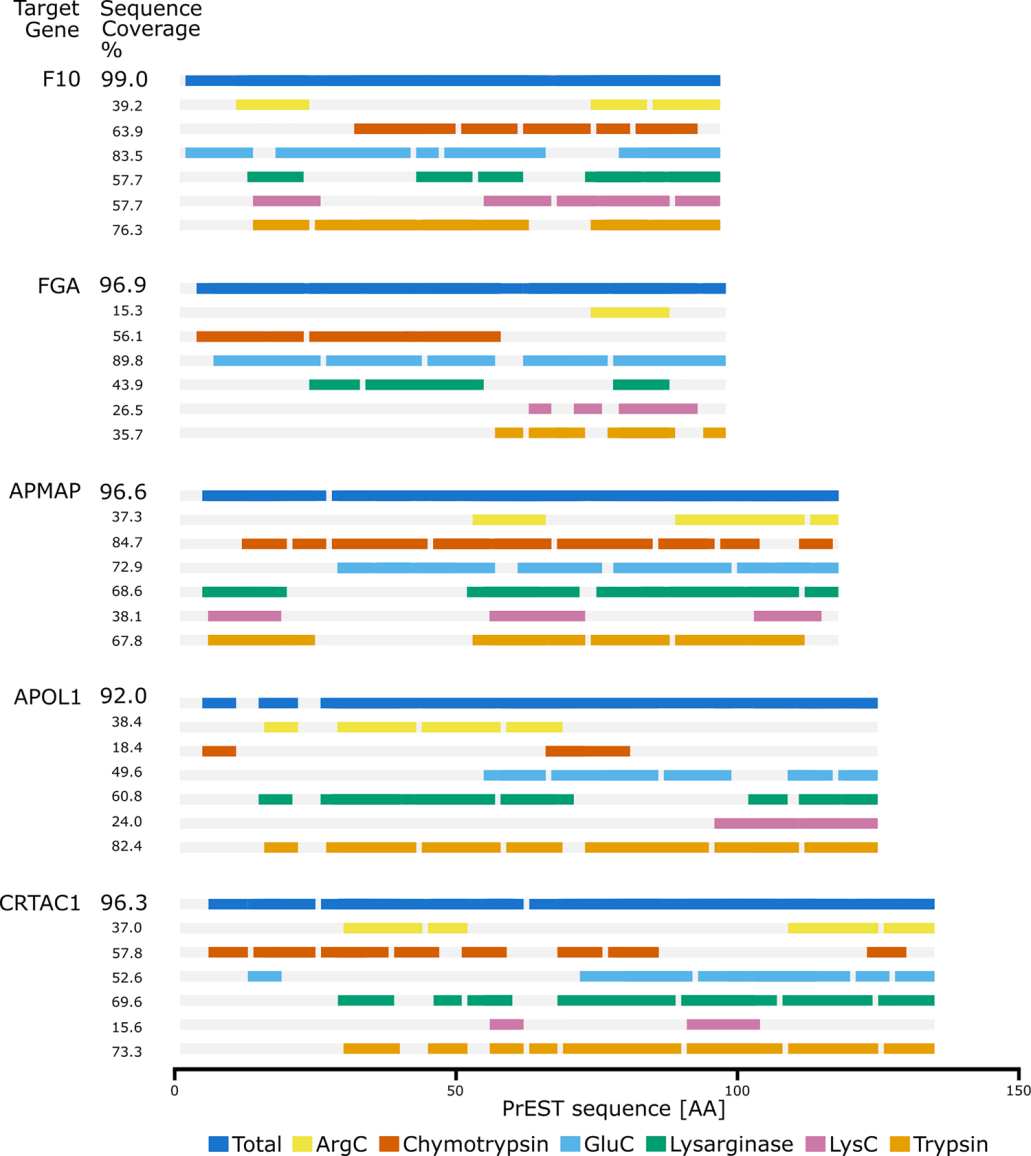
Sequence coverage of recombinant protein standards. Peptides identified during the SRM assay development by six proteases with its PrEST sequence coverage.

We set out to investigate whether it is possible to overcome the previously described protease bias in targeted proteomics and utilize different proteases for absolute quantification in targeted proteomics workflows with recombinant protein standards. First, we wanted to explore multiple proteases as a tool for extending the protein sequence coverage of existing recombinant protein standard libraries. We developed and validated SRM assays for 10 plasma protein targets using recombinant protein standards, termed PrESTs, with the proteases ArgC, GluC, chymotrypsin, lysarginase, LysC, and trypsin (Figure 1 and Table 1). In total, we identified 557 peptides generated by six different proteases from 10 recombinant protein standards. ArgC identified 38, GluC 100, chymotrypsin 89, lysarginase 122, LysC 70, and trypsin 138 peptides. The peptide coverage corresponded to a mean sequence coverage of 95.1% (Figures 2 and S1). This was a 23% increase on average, compared to the 77.3% tryptic peptide coverage alone. When comparing the sequence coverage of the six different proteases, we could observe that proteases that do not cleave at lysine or arginine extended the tryptic sequence coverage the furthest. The combination of trypsin with GluC and chymotrypsin achieves the highest overall sequence coverage when using only three proteases (Table 2). This suggests that alternative proteases have the possibility of extending the use of recombinant protein or fragment standard libraries, not only in terms of coverage but also to include quantitative assays from regions not covered by tryptic peptides.

**Table 2:** Top 20 Combinations of Up To Three Proteases That Achieved the Highest Total Sequence Coverage on PrESTs

protease 1	protease 2	protease 3	total sequence coverage [%]
GluC	trypsin	chymotrypsin	93.1
GluC	lysarginase	chymotrypsin	92.2
trypsin	lysarginase	chymotrypsin	91.7
trypsin	LysC	chymotrypsin	91.0
trypsin	ArgC	chymotrypsin	90.8
trypsin	chymotrypsin		90.2
GluC	trypsin	lysarginase	90.0
GluC	trypsin	LysC	89.2
GluC	ArgC	chymotrypsin	89.2
GluC	LysC	lysarginase	89.0
GluC	trypsin	ArgC	88.8
LysC	lysarginase	chymotrypsin	88.7
GluC	trypsin		88.2
GluC	ArgC	lysarginase	87.8
GluC	LysC	ArgC	87.6
GluC	LysC	chymotrypsin	86.7
GluC	lysarginase		86.5
LysC	ArgC	chymotrypsin	86.4
ArgC	lysarginase	chymotrypsin	85.4
trypsin	LysC	lysarginase	84.6

**Figure 3:**
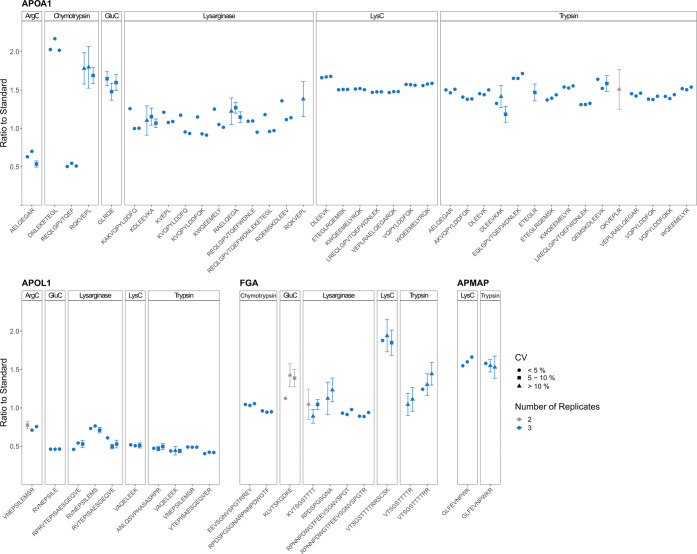
Peptide levels in plasma digest quantified with SIS-PrESTs. The mean ratio to standard of each digestion replicate included for each peptide. The ratio to standard calculated across replicates and visualized with error bars (±1 SD, bars not visualized if the technical CV is lower than 5%). Square dots (5–10% CV), triangle dots (>10% CV), blue dots (three replicates), and gray dots (two replicates).

After establishing SRM assays with six different proteases for 10 recombinant protein standards, we set out to quantify the selected target proteins in human plasma. All established SRM assays were evaluated in EDTA plasma, and only peptides passing the evaluation steps described in the Methods section were included in the following quantification. We spiked seven SIS-PrESTs to plasma at concentrations close to their respective endogenous levels to enable precise quantification on an absolute scale. The following quantification analysis included only peptides with a clear heavy signal. By applying further strict quality control measures, by only including peptides with a dotp and rdotp value equal to or larger than 0.9 in Skyline and a maximum CV of 20% between the ratio to standard of the triplicate injections, we were able to quantify peptides using at least two proteases covering the proteins APMAP, APOA1, APOL1, and FGA (Figure 3). A total of 60 peptides of six different proteases passed the quality control, which highlights the importance of the stringent SRM assay development for robust peptide quantification assays. The mean ratio to standard between the recombinant protein standard and endogenous protein ranged from 0.4 to 2.2, highlighting the accuracy of the spiked SIS-PrESTs. The majority of peptides displayed CVs below 5% between injection replicates. The quantified peptides of these plasma proteins spanned 3 orders of magnitude (0.02–70 μM).

**Figure 4:**
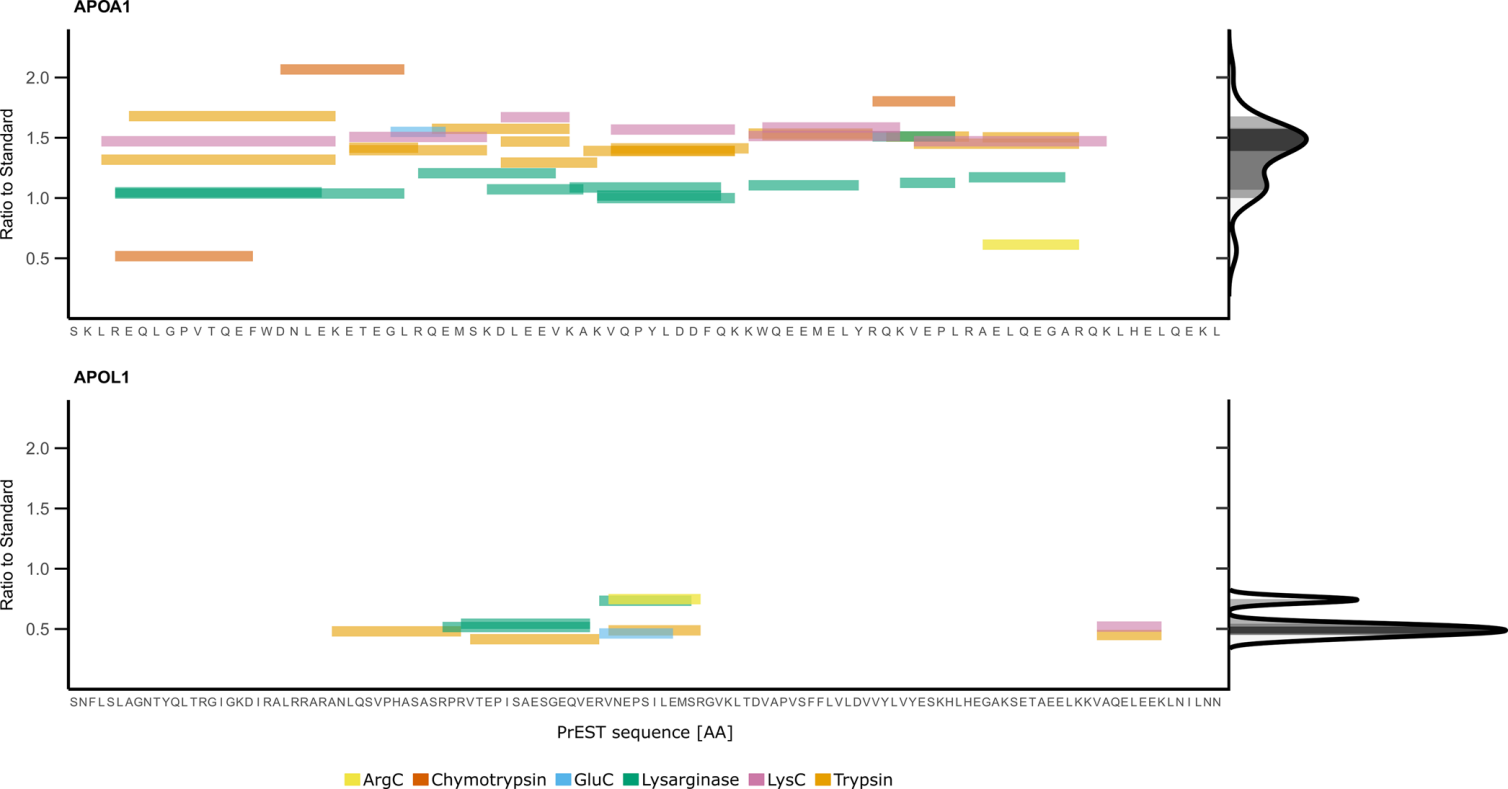
Absolute quantification of plasma proteins using six proteases and SIS-PrESTs. Ratio to standard of all peptides quantified by six proteases on APOA1 and APOL1. The displayed ratio to standard is the mean ratio to standard of digestion triplicate. The distribution of peptides on APOA1 and APOL1 in regard to their ratio to standard is illustrated by the Gaussian kernel density estimation. The density of the ratio to standard is visualized on the right side. The gray shade highlights the peptide distribution that is within one of three different density cutoffs from the peak; dark (≤50% inclusion of all quantified peptides), medium (≤75% inclusion of all quantified peptides), and light (≤90% inclusion of all quantified peptides).

We compared the determined quantification results of six different proteases for each of the four plasma proteins. One would expect similar quantification results of peptides generated by the different proteases given that the sample and spike-in ratios were identical. Interestingly, quantified peptides of one protein, namely, APOA1, showed inconsistent quantification with up to 4-fold difference (APOL1 1.8-fold, FGA 2.1-fold, APMAP no fold change). However, some peptides can be considered quantitative outliers, despite being highly reproducible. It is important to note that 50% of the peptides of APOA1 with the lowest distance to each other display a ratio to standard between 1.39 and 1.57 (1.13-fold) (Figure 4). Two proteases, ArgC and lysarginase, deviate in parts in their quantitative accuracy if compared to the four other enzymes, without showing the same trend for the other protein targets. This suggests that it would be possible to validate the quantitative accuracy for peptides representing the protein level by using multiple peptides generated by different proteases and at the same time also increasing the peptide coverage of a target protein.

Overall, the quantified peptides displayed concordant quantitative results and show no significant differences at the global scale for four proteases (Figure S2). However, as mentioned above, peptides of lysarginase differed significantly from LysC and trypsin in one protein, namely, the highest abundant APOA1. Interestingly, no difference could be observed for peptides of the same protease when quantifying the lower abundant proteins (APOL1, FGA). This could suggest protease-specific performance biases that still deliver precise quantification but with insufficient accuracy. The results show that quantification based on a single peptide or single protease can be misleading unless this peptide has been thoroughly validated against other peptides from the target protein. Here, the use of different proteases for the validation is especially interesting as it compensates for digestion biases and can thereby identify potential quantitative outliers.

**Figure 5:**
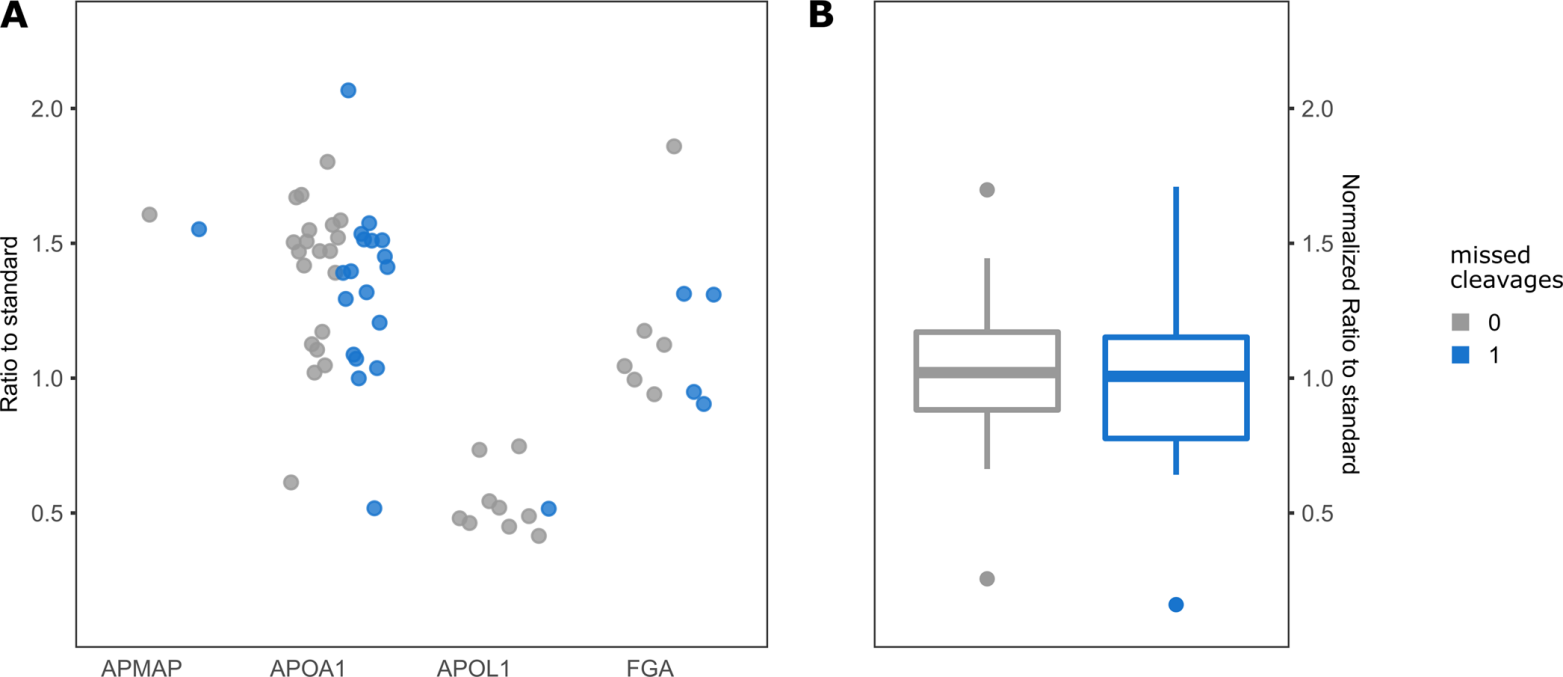
Comparison of peptides with non- to single miscleavages. (A) The mean ratio to standard of each peptide was included for quantification on APMAP, APOA1, APOL1, and FGA. Peptides with miscleavages are indicated in blue and those with no miscleavages are indicated in gray. (B) Boxplot displaying the mean centered ratio to the standard of all peptides shown in panel (A). The mean ratio to the standard of all peptides of one protein was normalized to 1. Median and IQR are displayed by box, and 1.5× IQR for outliers is illustrated by whiskers. *p*-Value of 0.48 was calculated by the unpaired Wilcoxon rank sum test between non- to single miscleavage peptides.

To further validate the peptide-specific performance of the six proteases, we also included miscleaved peptides in this study. We observed high concordance of their quantification results with the fully cleaved peptides. When comparing the mean centered ratio to standard between miscleaved and fully cleaved peptides, no significant bias in quantitative performance could be observed (*p*-value of 0.48) (Figure 5). We therefore suggest that miscleaved peptides should be considered for the quantification of endogenous proteins after thorough assay validation. This approach provides another way to extend the quantitative peptide repertoire of recombinant protein standards due to similar digestion kinetics of both endogenous protein and heavy labeled standards.

**Figure 6:**
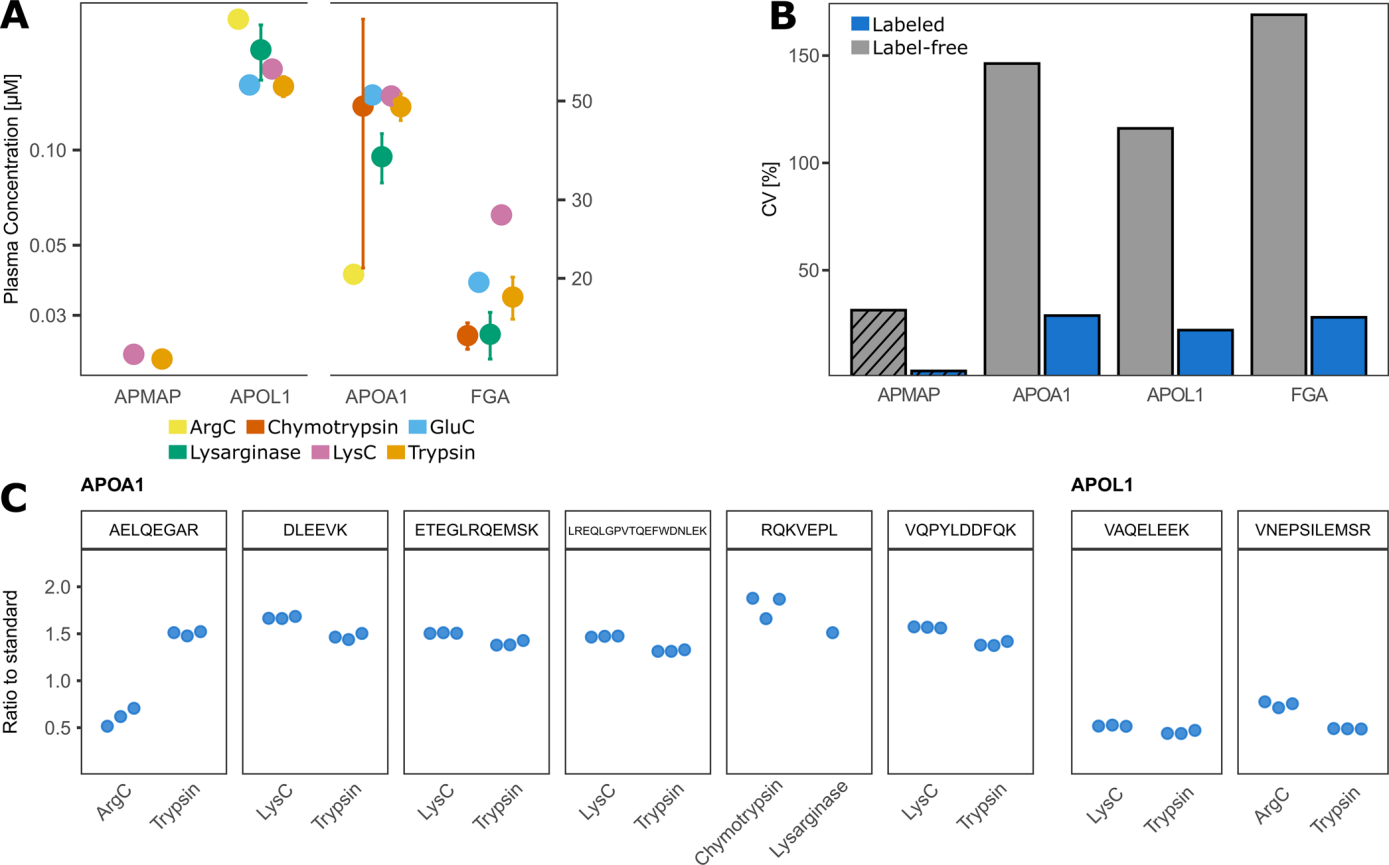
Variation in the absolute quantification of plasma proteins. (A) Mean endogenous concentration (±1 SD) of all peptides of one protease. Some proteases only contain one peptide for quantification. (B) Comparison of CVs between fragment peak area and concentration determined based on the heavy to light ratio of all peptides quantified on the protein. Striped if CV was determined using less than three proteases. (C) Comparison of the ratio to the standard of peptides that can be found in the digest of two different proteases. The median of each digestion replicate is shown.

Previous studies have highlighted that the use of different proteases influences the determined concentration of proteins in label-free quantification. To further investigate this bias, we examined the endogenous peak areas from proteins ranging from 0.02 to 70 μM (Figure 6A) by comparing all peptides within one protein to each other. Here, the variation between peptides identified was drastically increased in comparison to the variation in quantification based on recombinant protein standards (Figure 6B). The observed CVs between the peak areas of the target proteins range from 30.9 to 169.5%. CVs based on the absolute quantification by SIS recombinant protein standards were lower spanning from 2.4 to 28.4%. Therefore, we support the previously observed protease bias in label-free quantification and demonstrate that the protease bias can be observed in label-free SRM assays. We can report that the protease bias is reduced by including recombinant protein standards in the experimental setting. However, it is important to note that the variation between specific single peptides was still up to fourfold and further investigation in regard to PTMs, protein isoforms, or protein structures influencing the quantification as well as protease performance has to be made. It also has to be noted that the variation between peptides of one protease was for most parts lower than that between peptides of different proteases. Therefore, the protease specificity, as well as structural accessibility of the protein sequence, could still play a role in the observed quantitative variation.

To further address the quantification consistency between different proteases, we focused on identical peptides independently generated by different proteases. Due to similar cleavage sites of certain proteases, they generated the same peptides in plasma by different proteases. We quantified eight identical peptides that cover the proteins APOA1 and APOL1. Seven peptides showed similar quantitative performance in both protease digests (Figure 6C). Despite some variation between different proteases, the observed variation was lower for technical replicates. These quantifications were performed in different peptide matrices and therefore further highlight the strength of using multiple proteases in combination with recombinant protein standards to validate the quantitative accuracy. At the same time, visible biases between the same peptides of different proteases suggest that digestion conditions, protein structure, and the enzyme itself could potentially influence the protein quantification even on an absolute scale. Whether this also holds true for the application of peptide standards has yet to be shown.

## Conclusions

In this study, we explored the application of alternative proteases for absolute quantification in targeted proteomics. Previous work described a quantification bias between multiple proteases in bottom-up proteomics. We show that recombinant protein standards provide a way of reducing the previously highlighted issue of protease bias in targeted mass spectrometry. The presented work highlights the strength of multiple protease approaches not only for extended protein coverage but more so for the accurate quantification of protein in targeted MS. This quantitative strategy could unlock the possibility to evaluate the quantitative performance on an absolute scale, which is needed for clinical tests used for diagnostics applications. This study has evaluated the quantitative accuracy when quantifying blood plasma proteins using different proteases and shows the strength of the multiprotease strategy. However, the choice of protease could affect the quantitative accuracy, which has to be carefully evaluated when selecting protein targets. With this, we suggest the application of multiple proteases as an easy-to-access and novel strategy for in-depth validation of the quantitative performance of peptides when absolute quantification is needed.

## Supporting Information Available

Peptides identified on five PrESTs by six proteases included in the SRM assay (Figure S1) and comparison of protease bias on the normalized ratio to the standard of quantitative peptides (Figure S2) (PDF)
