# Supplementary material for: Addressing the Protease Bias in Quantitative Proteomics
Source: J Proteome Res. 2022 Aug 31;21(10):2526–34. doi: 10.1021/acs.jproteome.2c00491 (PMC9552229; doi:10.1021/acs.jproteome.2c00491)
Supplement: Supplementary file 1 — pr2c00491_si_001.pdf [file pr2c00491_si_001.pdf]

Supporting Information

Addressing the protease-bias in quantitative proteomics

Jakob Woessmann[1,2], David Kotol[1,2], Andreas Hober[1,2], Mathias Uhlén[1,2], Fredrik Edfors* [1,2]

1. Science for Life Laboratory, KTH - Royal Institute of Technology, 17165 Solna, Sweden
2. Department of Protein Science, KTH - Royal Institute of Technology, 10691 Stockholm, Sweden

*Corresponding author. Email: fredrik.edfors@scilifelab.se

Table of contents

Supplementary Figures

Figure S1 Peptides identified on five PrESTs by six proteases included in the SRM assay.

Figure S2 Comparison of protease bias on normalized ratio to standard of quantitative peptides.

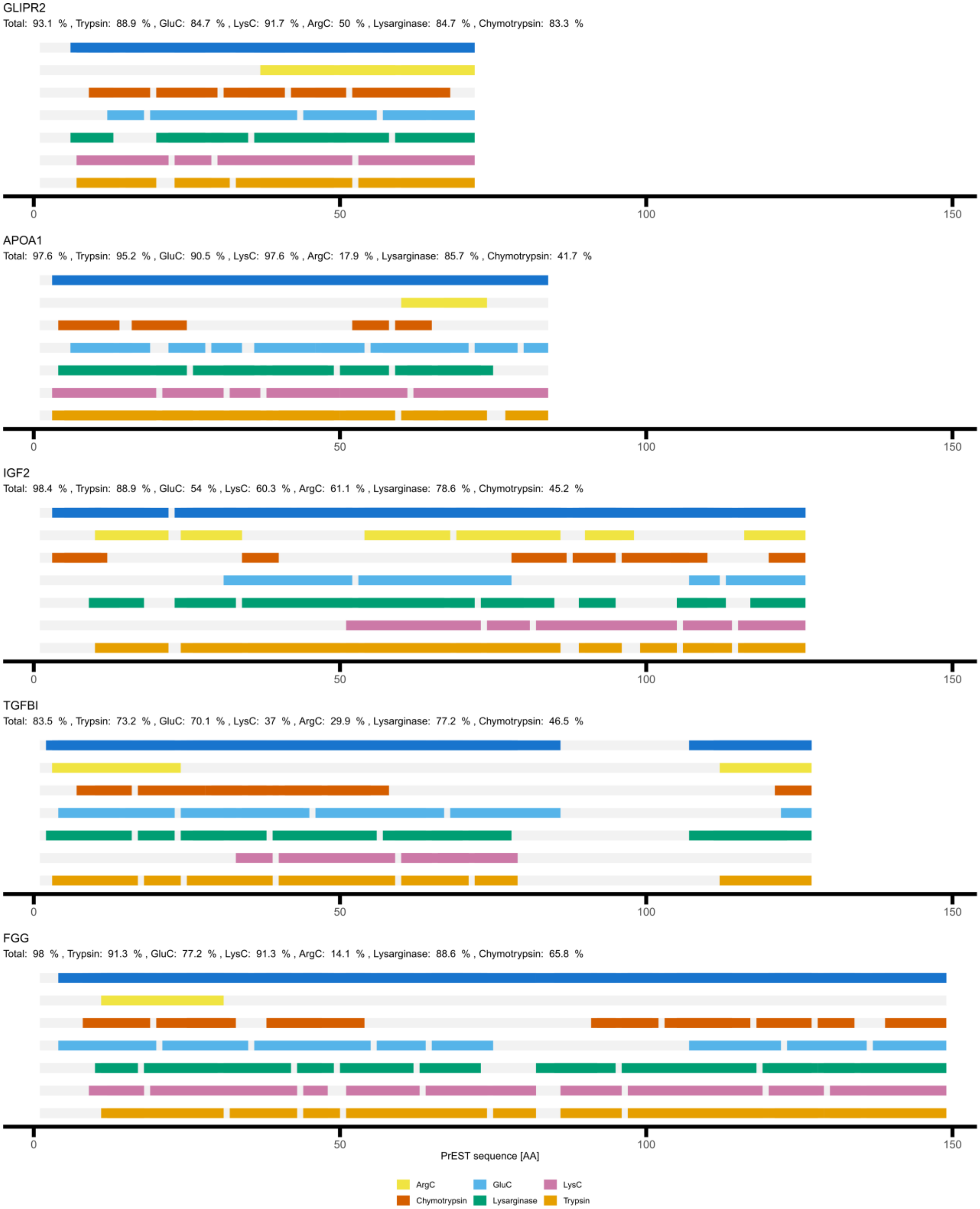

Figure S1: Peptides identified on five PrESTs by six proteases included in the SRM assay. PrEST sequence coverage by peptides of six proteases included in the developed SRM assays. Percentage sequence coverage was calculated for each protease and for the total PrEST sequence.

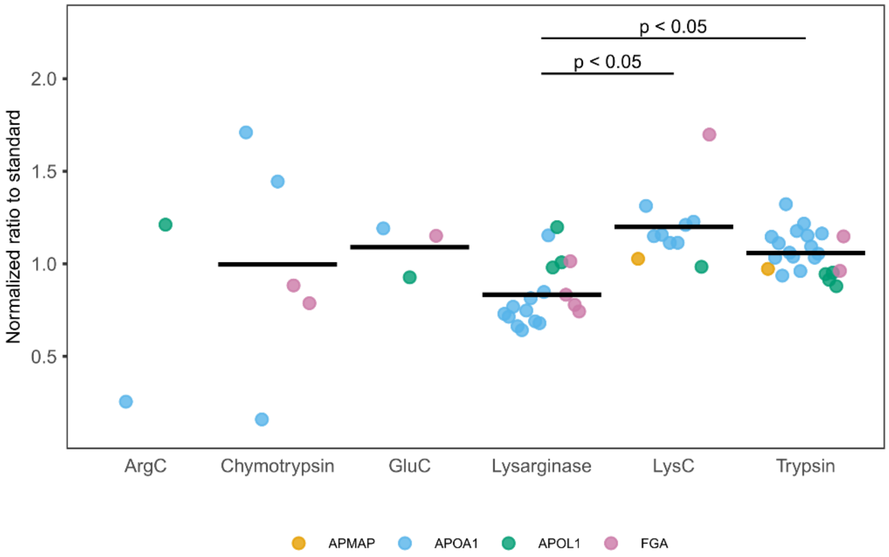

Figure S2: Comparison of protease bias on normalized ratio to standard of all quantitative peptides. The mean ratio to the standard of all peptides of one protein was normalized to 1. Mean of normalized ratio to standard of peptides for each protease shown. Statistical evaluation of quantitative variation between proteases evaluated by Pairwise Wilcoxon Rank Sum Tests corrected by Holm and p-values below 0.05 shown.
